# *In vitro* growth of *Plasmodium falciparum* in neonatal blood

**DOI:** 10.1186/1475-2875-13-436

**Published:** 2014-11-18

**Authors:** Ulrich Sauerzopf, Yabo J Honkpehedji, Ayôla A Adgenika, Elianne N Feugap, Ghyslain Mombo Ngoma, Jean-Rodolphe Mackanga, Felix Lötsch, Marguerite M Loembe, Peter G Kremsner, Benjamin Mordmüller, Michael Ramharter

**Affiliations:** Department of Medicine I, Division of Infectious Diseases and Tropical Medicine, Medical University of Vienna, Vienna, Austria; Centre de Recherches Médicales de Lambaréné, Hôpital Albert Schweitzer, Lambaréné, Gabon; Institut für Tropenmedizin, Universität Tübingen, Wilhelmstraße 27, 72074 Tübingen, Germany; Department of Parasitology, Leiden Medical University Center, Leiden, The Netherlands; Faculté de Médecine et des Sciences de la Santé, Université Omar Bongo, Libreville, Gabon

**Keywords:** *Plasmodium falciparum*, Neonatal haemoglobin, Malaria, Histidine-rich protein 2, Immune plasma

## Abstract

**Background:**

Children below the age of six months suffer less often from malaria than older children in sub-Saharan Africa. This observation is commonly attributed to the persistence of foetal haemoglobin (HbF), which is considered not to permit growth of *Plasmodium falciparum* and therefore providing protection against malaria. Since this concept has recently been challenged, this study evaluated the effect of HbF erythrocytes and maternal plasma on *in vitro* parasite growth of *P. falciparum* in Central African Gabon.

**Methods:**

Umbilical cord blood and peripheral maternal blood were collected at delivery at the Albert Schweitzer Hospital in Gabon. Respective erythrocyte suspension and plasma were used in parallel for *in vitro* culture. *In vitro* growth rates were compared between cultures supplemented with either maternal or cord erythrocytes. Plasma of maternal blood and cord blood was evaluated. Parasite growth rates were assessed by the standard HRP2-assay evaluating the increase of HRP2 concentration in Plasmodium culture.

**Results:**

Culture of *P. falciparum* using foetal erythrocytes led to comparable growth rates (mean growth rate = 4.2, 95% CI: 3.5 – 5.0) as cultures with maternal red blood cells (mean growth rate =4.2, 95% CI: 3.4 – 5.0) and those from non-malaria exposed individuals (mean growth rate = 4.6, 95% CI: 3.8 – 5.5). Standard *in vitro* culture of *P. falciparum* supplemented with either maternal or foetal plasma showed both significantly lower growth rates than a positive control using non-malaria exposed donor plasma.

**Conclusions:**

These data challenge the concept of HbF serving as intrinsic inhibitor of *P. falciparum* growth in the first months of life. Erythrocytes containing HbF are equally permissive to *P. falciparum* growth *in vitro*. However, addition of maternal and cord plasma led to reduced *in vitro* growth which may translate to protection against clinical disease or show synergistic effects with HbF *in vivo*. Further studies are needed to elucidate the pathophysiology of innate and acquired protection against neonatal malaria.

## Background

Although children below five years of age are disproportionally severely affected by malaria morbidity and mortality, there is a lag time of about three months after birth, before first disease episodes occur [1–4]. This period coincides with the persistence of maternal IgG antibodies in the infant’s circulation but other protective mechanisms may also contribute to the reduced susceptibility [4–7].

Previous studies postulate a role for persisting foetal haemoglobin during the first months of life in protection against malaria [8]. The transcription of HbF starts around the 10th week of development and ends shortly before birth. This causes a linear decline in the number of foetal red blood cells from up to 90% at time of birth to around 5% at 3 months of age [8]. It was hypothesized that parasite strains – encountering HbF only on relatively rare occasions from an evolutionary perspective – may therefore be selected by strong adaptation to adult haemoglobin (HbA), a process resulting in limited intra-erythrocytic growth of *Plasmodium falciparum* in predominantly HbF containing neonatal blood [9, 10]. This paradigm of protection during early infancy – first published in 1977 – was held up since then until recently, when Amaratunga *et al.* failed to detect growth delays in neonatal erythrocytes and thus profoundly challenged this concept [11].

To further investigate potential mechanisms of protection, this study evaluated the comparative *in vitro* growth rates of a standardized *P. falciparum* clone under controlled culture conditions using either maternal, cord, or non-malaria exposed donor erythrocytes.

In addition, the growth modulating effect of maternal, cord, and non-malaria exposed donor plasma on P. falciparum growth was evaluated.

## Methods

### Study region and patient population

The study took place at the Centre de Recherches Médicales de Lambaréné, Albert Schweitzer Hospital and Georges Rawiri Regional Hospital in Lambaréné, Gabon. Gabon is a Central African country characterized by a tropical climate and hyperendemic malaria transmission [12]. Participants were invited to join this study when attending the local maternity wards for delivery. Demographic data were obtained, however no validated information about intake of IPTp was available. All subjects were tested in routine antenatal care for HIV and only those with negative test result were invited to participate. The study protocol was approved by the institutional review board of the CERMEL and the ethical review committee of the Medical University of Vienna and all women provided written informed consent prior to blood sampling.

All samples were tested for sickle cell trait by haemoglobin electrophoresis and for plasmodial infection by thick smear at the time of delivery and samples were excluded in case of a positive result or when signs of haemolysis or clotting were apparent. At delivery 1.2 ml of blood was taken from the umbilical cord in an EDTA tube. Peripheral maternal venous blood was taken at the earliest convenience within three days. Non-malaria exposed donor blood was obtained from Caucasian male volunteers. Blood samples were collected in EDTA tubes and specimens were immediately centrifuged to separate red blood cells from plasma. Red blood cells were washed three times in complete parasite medium and stored at 4°C in 0.5 volume of saline adenine glucose-mannitol (150 mM NaCl, 1.25 mM adenine, 45 mM glucose, 30 mM mannitol) until further use. Plasma was immediately frozen at -80°C.

### Parasite culture and growth assay

A laboratory-adapted clone of *P. falciparum* (3D7) repeatedly selected for presence of knob-phenotype was kept in continuous sorbitol synchronized culture throughout the course of the study. Parasites were maintained using a standard protocol in complete parasite medium (500 ml RPMI-1640, 10 mg/l gentamicin, 6 g/l (25 mM) HEPES, 292 mg/l (2 mM) L-glutamine, 50 mg/l (0.36 mM) hypoxanthine, 5 g/l Albumax II) in a candle jar at 37°C. To test the effect of erythrocytes and plasma on plasmodial growth, micro-cultures of 200 μl each were established in 96-well plates (Corning Costar-3599) in duplicates. To investigate the effect of HbF on parasite growth, micro-cultures containing cord derived red blood cells and maternal red blood cells, respectively, as well as a positive control containing donor O + red blood cells, were set up in duplicates. Parasites derived from continuous culture in adult RBC were diluted with respective test RBC (>1 : 40) to obtain at 1.5% haematocrit, 0.05% parasitaemia using 5% pooled, heat inactivated serum from AB + donors for 72 hours before growth was stopped by freezing.

Plasma derived from mothers, newborns and a malaria-naïve adult was added to cultures in complete parasite medium to assess its effect on parasite growth at 1.5% haematocrit, 0.05% parasitaemia and 10% plasma supplementation. Frozen lysates of micro-cultures were used for histidine-rich protein II enzyme linked immunosorbent assay (HRP2 ELISA) for the quantification of parasite growth [13–15]. Commercial antibodies (MPFM-55A and MPFG-55P, Immunology Consultants Laboratories, Inc) and high binding plates (Corning Costar 3590) were used. Growth rates of *P. falciparum* were estimated by the assessment of HRP2 concentrations in cultures. Growth was defined as an increase in optical density in the HRP2 ELISA. An at least two fold increase in HRP2 concentration from prior to post incubation was set as threshold for further analysis. Statistical analysis was performed using “R” software. Differences between groups were assessed employing the Tukey's Honestly Significant Difference test. All reagents, unless stated otherwise were obtained from Sigma-Aldrich, St. Louis.

## Results

Hundred and three pregnant women consented to participate in this study between July 2012 and June 2013, of which sixty-five were excluded from further testing (sickle cell trait: 12, clotting: 27, haemolysis after sampling: 13, Plasmodium infection: 7, presence of anti-Rhesus antibodies: 6). Thirty-eight participants finally met the criteria for analysis. Mother's age was ranging from 17 to 42 years with a mean of 25 years and a standard deviation of 7.1 years with a mean gravidity of 3.5 with a standard deviation of 2.4. Mean gestational age at delivery was 38.5 weeks of gestation with a standard deviation of 2.1 weeks and mean birth weight was 2,866 grams ranging from 1,900 to 4,400 grams. Among the recruited women, 18 paired erythrocyte samples and 22 plasma specimens were successfully used in parallel for further *in vitro* testing.

### Maternal, neonatal, and non-malaria exposed donor erythrocytes

**Figure 1:**
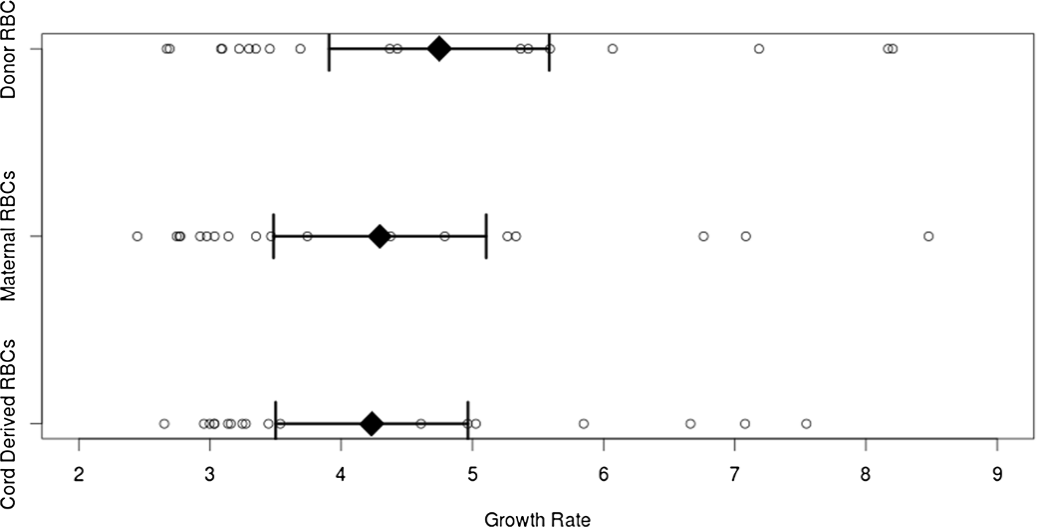
***In vitro*** **growth rates of** ***Plasmodium falciparum*** **when using cord, maternal or non-malaria exposed donor erythrocytes displayed by data points, means and 95% CIs.**

Parasite growth was quantified by HRP2 ELISA. 18 paired samples of maternal, cord and donor RBCs showed adequate growth and were used for comparative evaluation. Mean cumulative growth rates were 4.35 (95% confidence interval: 3.90 – 4.81) folds when compared with pre-incubation samples. No significant difference between cultures using non-malaria exposed donor erythrocytes (mean growth rate: 4.6, 95% CI: 3.8 – 5.5) or either maternal (4.2, 95% CI: 3.4 – 5.0) or cord derived erythrocytes (4.2, 95% CI: 3.5 – 5.0) was observed (Figure 1). Similarly, growth rates between maternal and cord derived erythrocytes supplemented cultures did not differ (p = 0.99). To test these findings under different culture conditions, cultures were classified in high growth (>4 fold increase) and low growth strata (2–4 fold increase in HRP2). When comparing growth rates within these strata, again no significant difference was observed (Table 1).

**Table 1:** **Mean growth rates and lower and upper margin of 95% CIs in test given test erythrocytes of high and low growth strata as well as the cumulative data**

All data	Lower margin	Mean	Upper margin
Cord derived RBC	3.50	4.23	4.97
Maternal RBC	3.38	4.19	5.00
Non malaria exposed donor RBC	3.79	4.63	5.47
**Low growth**			
Cord derived RBC	4.63	5.36	6.08
Maternal RBC	4.63	5.43	6.23
Non malaria exposed donor RBC	5.42	6.09	6.76
**High growth**			
Cord derived RBC	3.01	3.11	3.21
Maternal RBC	2.81	2.95	3.10
Non malaria exposed donor RBC	3.02	3.17	3.33

**Figure 2:**
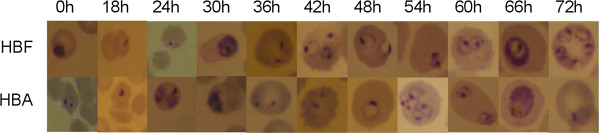
***In-vitro*** **development of** ***Plasmodium falciparum*** **over time in erythrocytes rich in foetal and adult haemoglobin respectively.**

In addition to this quantitative analysis, a qualitative analysis of intra-erythrocytic growth of *P. falciparum* was performed microscopically. Parasites growing in umbilical cord erythrocytes displayed microscopically similar maturation characteristics and no other obvious morphological differences were observed (Figure 2).

### Maternal, neonatal, and non-malaria exposed plasma

**Figure 3:**
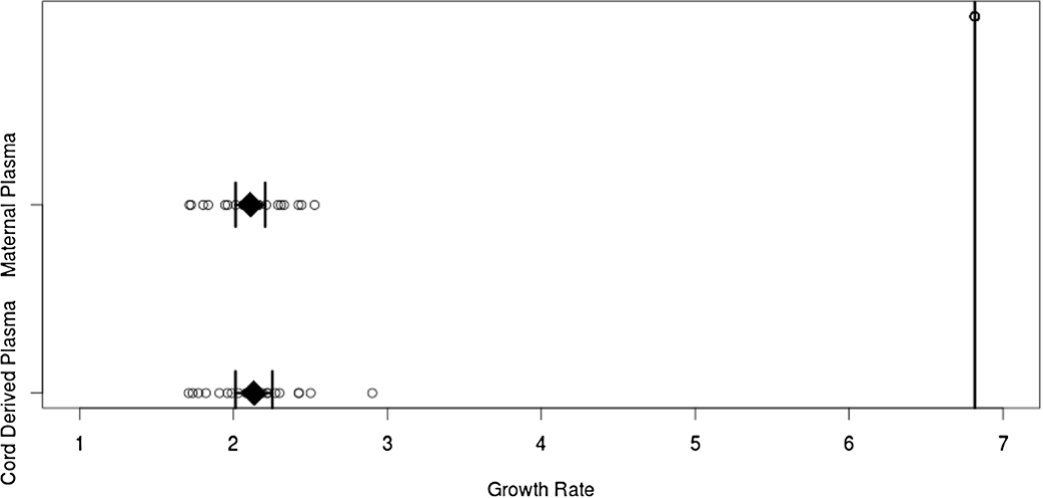
***In vitro*** **growth rates of** ***Plasmodium falciparum*** **when supplemented with either cord or maternal plasma displayed by data points, means and 95% CIs.** Non-malaria exposed donor plasma used as a positive control yielded a growth rate of 6.8, indicated by the vertical bar.

Growth rates of *P. falciparum* were compared under standard conditions by supplementing culture medium with either maternal or cord derived plasma and non-malaria exposed donor plasma serving as a positive control. *Plasmodium falciparum* growth was significantly higher in non-malaria exposed donor plasma containing culture medium than in either culture supplemented with test plasma (Figure 3). Whereas the mean increase in HRP2 concentration in culture supplemented with malaria-naïve plasma was 6.8 fold, growth rates in cultures supplemented with maternal and umbilical cord plasma averaged 2.1 fold (p <0.001). Umbilical cord plasma (mean growth rate: 2.1, 95% CI: 2.0 - 2.3) and maternal plasma (mean growth rate: 2.1, 95% CI: 2.0 - 2.2) yielded close to identical results. (p =0.93)

## Discussion

This study demonstrates that cord derived erythrocytes, harbouring a high concentration of HbF, are equally suited for *P. falciparum* invasion, maturation, and growth as adult erythrocytes containing predominantly HbA. Growth rates between maternal, neonatal and non-malaria exposed donor erythrocytes were very similar, and no morphological signs of growth inhibition were detected. These findings provide strong evidence that *P. falciparum* may similarly develop within HbF rich red blood cells and that HbF per se is no protective factor against malaria during the first months of life.

These data are in line with results published by Amaratunga *et al.* [11] demonstrating normal invasion and development of *P. falciparum* in HbF containing red blood cells in a set of adult and six cord blood specimens. While this study focused on an overall assessment of parasite growth in a larger sample size, Amaratunga *et al.* could detect effects of neonatal haemoglobin, in particular in conjunction with immune IgGs, on the knob phenotype and PfEMP-1 mediated cytoadherence of parasitized red blood cells. Although erythrocytes rich in foetal haemoglobin seem to be permissive to infection with *P. falciparum in vitro*, reduced cytoadherence may, particularly in the presence of IgG, lead to lower parasite densities *in vivo* due to increased clearance of infected cells in the spleen.

To evaluate whether humoral immunity and transfer of maternal immunoglobulins or other plasmatic factors may play a protective role in newborns, cultures were supplemented with the respective plasma samples. Interestingly, a consistent growth-inhibition was observed in both maternal and neonatal plasma when compared to non-malaria exposed donor plasma. *In vitro* growth inhibition caused by a supplementation with immune plasma was reported previously in Thai individuals yielding comparable results to this study [16].

It is tempting to speculate that passive transfer of maternal antibodies to the newborn accounts for this growth inhibitory effect. However, it may not be excluded that other factors including cytokines, hormones, complement or indeed drugs with anti-malarial activity passing the placental barrier into the neonatal blood may have caused this inhibition of growth. One limitation of this study is the lack of valid information about IPTp intake of study participants – a factor potentially affecting plasma assays. In addition these data derive from experiments with one clone of *P. falciparum* and potential differences between wild isolates and laboratory adapted parasites may not be ruled out. Finally, behavioural factors certainly also contribute to young infants’ protection from malaria, since neonates receive special care and attention by their caregivers potentially leading to less exposure to infectious bites.

The amount of protection against malaria in early infancy might yet be caused by the interplay of several distinct factors, probably in conjunction with maternally derived antibodies. However, these data conclusively refute the hypothesis of HbF serving as an intrinsic inhibitor of *P. falciparum* growth *in vitro*, and indicate that maternal and newborn plasma show considerable inhibitory activity against *in vitro* growth of *P. falciparum*.
